# Spontaneous resolution of an inferior epigastric artery pseudoaneurysm secondary to surgical drain placement

**DOI:** 10.1308/003588412X13373405384855

**Published:** 2012-09

**Authors:** JS Williamson, RL Harries, G Davies, A Woodward

**Affiliations:** Cwm Taf Health Board,UK

**Keywords:** Pseudoaneurysm, Epigastric arteries, Disease management

## Abstract

We report the case of a 73-year-old woman who was found to have an inferior epigastric artery pseudoaneurysm caused by surgical drain placement during a laparotomy for an appendix abscess. She presented with pain around the drain site two months following surgery and intravenous contrast computed tomography (CT) revealed a right inferior epigastric artery pseudoaneurysm. A decision was made to manage this expectantly as she remained stable with no other complications. Following a period of nine months of observation, repeat intravenous contrast CT showed evidence of spontaneous regression and thrombosis of the pseudoaneurysm. She remains well and her symptoms have now resolved.

## Case history

A 73-year-old Caucasian woman presented with right iliac fossa pain and clinical evidence of sepsis three months following an admission with an appendix abscess that had been treated non-operatively with intravenous antibiotics. Her previous medical history included hypertension, asthma, an open cholecystectomy and bladder cancer diagnosed two years previously. She was a non-smoker with no significant family history of note. Clinical examination revealed tenderness in the right iliac fossa associated with pyrexia and tachycardia as well as an elevated white blood count (19.6 × 10^9^/l) and C-reactive protein level (208mg/l). Computed tomography (CT) of the abdomen and pelvis revealed several loculated pelvic collections of varying size between 2cm and 5cm in their longest axes that were not amenable to percutaneous drainage, associated with evidence of an inflamed appendix.

As conservative management had failed, a midline laparotomy was performed the day following admission. This revealed multiple loculated interloop abscesses with an inflamed appendix that was adherent to the pelvic floor. The abscesses were drained and irrigated with saline and an appendicectomy was performed with oversewing of the stump using Vicryl® (Ethicon Inc, Somerville, NJ, US) 3/0 sutures. A 20Fr Portex® Robinson surgical drain (Smiths Medical, Watford, UK) was sited in the right iliac fossa. This was inserted by making a skin crease incision in the anterior abdominal wall at the right iliac fossa and then, using a Robinson drain, introducing a trocar to pierce the underlying fascia, muscle layers and peritoneum of the anterior abdominal wall under direct vision. No bleeding was observed.

Post-operative recovery was uneventful. The drain output was initially 150ml per day for the first three days, then reduced to 60ml per day. On day 6, it was commented that the drain output appeared purulent and it was therefore left in situ at this time. In the days following this, the drain output became clear and reduced in volume every day. As the patient remained well with no evidence of undrained sepsis, the drain was removed after 11 days. There was no pain, bleeding or other complications noted at this time. The patient was discharged home on day 16 after admission.

**Figure 1:**
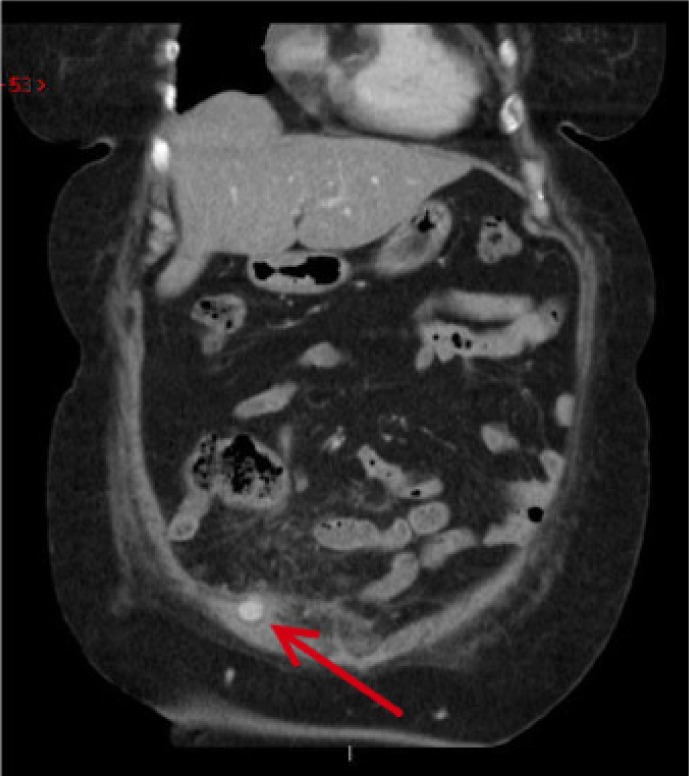
Intravenous contrast computed tomography revealing right inferior epigastric artery pseudoaneurysm (arrow)

Histological examination of the appendix revealed numerous inflammatory cells with no evidence of dysplasia or malignancy. Two months later on review in the outpatient clinic, the patient complained of a burning pain in the right iliac fossa and around the previous drain site that had started several weeks following the surgery. On examination, there was tenderness in the right iliac fossa but no evidence of a pulsatile mass around the drain site and the wound had healed well. Repeat intravenous contrast CT was requested to exclude a recurrent collection and this revealed a 1cm pseudoaneurysm of the right inferior epigastric artery, which was not present on the pre-operative CT (Fig 1).

Opinions were sought from a consultant vascular surgeon and a consultant interventional radiologist regarding further management. The decision was taken to manage expectantly without any specific intervention unless there was any deterioration as the patient’s symptoms were controlled with simple analgesia.

**Figure 2:**
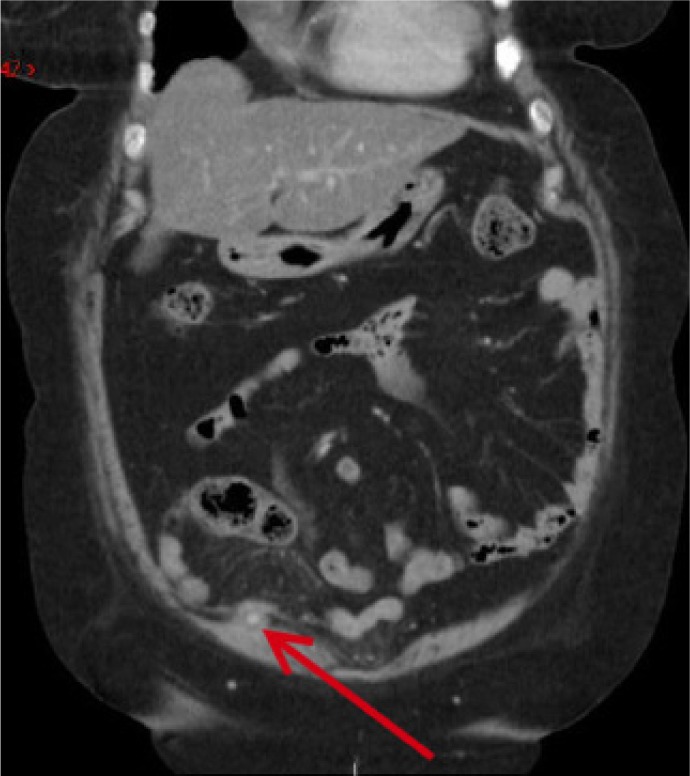
Intravenous contrast computed tomography revealing partial resolution of inferior epigastric artery pseudoaneurysm at nine months

Follow-up CT performed nine months later showed that the right inferior epigastric artery pseudoaneurysm had thrombosed (Fig 2). The patient remains well and her symptoms have now resolved fully.

## Discussion

The inferior epigastric artery is susceptible to injury during abdominal wall procedures due to its position in the rectus sheath. However, despite this, pseudoaneurysms of the inferior epigastric artery are rare. Reported cases in the literature have been associated with trauma, removal of retention sutures, percutaneous biopsy, paracentesis, Tenckhoff catheter removal and formation of stoma.^1^ There has been one other reported case of inferior epigastric artery pseudoaneurysm formation secondary to surgical drain placement.^2^ In this case, the pseudoaneurysm presented acutely with right iliac fossa pain, large abdominal wall haematoma and anaemia 48 hours post-operatively. The authors also managed the pseudoaneurysm conservatively, allowing the tamponade effect of the associated rectus sheath haematoma to compress the pseudoaneurysm, although no subsequent follow-up information was reported.

Various other treatment options have been reported including open surgical ligation, ultrasonography guided percutaneous thrombin, coil embolisation^3^ and ultrasonography guided compression.^4^ In our case, the decision was made to reserve surgical or radiological management of the pseudoaneurysm and to adopt a ‘watch and wait’ approach as the patient remained stable and her symptoms were well controlled with simple analgesia. Furthermore, endovascular treatment has been associated with post-procedural pain, arterial thrombosis and failure.^2^ Spontaneous thrombosis of pseudoaneurysms has also been reported in varying locations in the body including splenic and hepatic arteries following trauma.^5^ We therefore emphasise that in those patients with inferior epigastric artery pseudoaneurysm who remain asymptomatic, conservative management can result in spontaneous regression and thrombosis. However, it should be noted that for large or rapidly growing pseudoaneurysms, or for those failing to resolve spontaneously, treatment options such as ultrasonography guided compression or percutaneous thrombin, coil embolisation and even surgical ligation should be considered.
